# hMOB2 deficiency impairs homologous recombination-mediated DNA repair and sensitises cancer cells to PARP inhibitors

**DOI:** 10.1016/j.cellsig.2021.110106

**Published:** 2021-11

**Authors:** Ramazan Gundogdu, M. Kadir Erdogan, Angeliki Ditsiou, Victoria Spanswick, Juan Jose Garcia-Gomez, John A. Hartley, Fumiko Esashi, Alexander Hergovich, Valenti Gomez

**Affiliations:** a Department of Biology, Bingol University, Bingol 12000, Turkey; b Department of Biochemistry and Biomedicine, University of Sussex, Brighton BN1 9QG, UK; c UCL Cancer Institute, University College London, London WC1E 6DD, UK; d Sir William Dunn School of Pathology, University of Oxford, Oxford OX1 3RE, UK; e Evotec France, Toulouse 31100, France

**Keywords:** DNA damage response signalling, Homologous recombination DNA repair, Mps one binder 2 (MOB2), Personalised PARP inhibitor treatments, MOB, Mps one binder, NDR, Nuclear Dbf2-related, DDR, DNA damage response, HR, homologous recombination, NHEJ, non-homologous end-joining, PARP, poly(ADP-ribose) polymerase, MRN, MRE11-RAD50-NBS1, IR, Ionising radiation, MMC, mitomycin C, DSB, DNA double-strand break, SSB, DNA single-strand break, ssDNA, single-stranded DNA, RNAi, RNA interference, TCGA, The Cancer Genome Atlas.

## Abstract

Monopolar spindle-one binder (MOBs) proteins are evolutionarily conserved and contribute to various cellular signalling pathways. Recently, we reported that hMOB2 functions in preventing the accumulation of endogenous DNA damage and a subsequent p53/p21-dependent G1/S cell cycle arrest in untransformed cells. However, the question of how hMOB2 protects cells from endogenous DNA damage accumulation remained enigmatic. Here, we uncover hMOB2 as a regulator of double-strand break (DSB) repair by homologous recombination (HR). hMOB2 supports the phosphorylation and accumulation of the RAD51 recombinase on resected single-strand DNA (ssDNA) overhangs. Physiologically, hMOB2 expression supports cancer cell survival in response to DSB-inducing anti-cancer compounds. Specifically, loss of hMOB2 renders ovarian and other cancer cells more vulnerable to FDA-approved PARP inhibitors. Reduced *MOB2* expression correlates with increased overall survival in patients suffering from ovarian carcinoma. Taken together, our findings suggest that hMOB2 expression may serve as a candidate stratification biomarker of patients for HR-deficiency targeted cancer therapies, such as PARP inhibitor treatments.

## Highlights

• hMOB2 promotes homologous recombination (HR) double-strand break (DSB) repair.
• hMOB2 is required for the stabilisation of RAD51 on damaged chromatin.
• Low hMOB2 levels potentiate the anti-tumour effects of the DNA-damaging agents.
• hMOB2 as a predictive biomarker to PARP inhibition and patient stratification.

## 1 Introduction

Human cells constantly receive genotoxic pressure from internal and external insults [1], [2]. The maintenance of genome integrity through well-coordinated DNA damage response (DDR) and repair mechanisms is required to protect cellular DNA from accumulating genomic instability, which is an essential hallmark of tumorigenesis [3]. Of the various types of cytotoxic damage, DNA double-strand breaks (DSBs) are defined as one of the most deleterious genomic lesions [1], [4], [5], [6]. Homologous recombination (HR) and non-homologous end joining (NHEJ) mechanisms are the two canonical pathways of DNA DSB repair [5], [6], [7], [8]. In contrast to the error-prone NHEJ mechanism that is functional throughout all cell cycle phases, the error-free HR mechanism is only operative in S-G2 cells where a homologous DNA template is available [1], [5], [7]. In addition to neoplastic transformation, defective DDR and DSB repair mechanisms can cause de novo and chemotherapy-acquired resistance [9], [10], [11], [12]. Therefore, extensive efforts have been devoted to targeting the DDR components for better treatment management [11], [12], [13], [14], [15], [16].

Poly(ADP-ribose) polymerases (PARPs) function as DNA damage sensors as they detect DNA single strand breaks (SSBs) and other types of lesions, and subsequently activate DNA repair machinery by transducing the damage signals to specific effectors through performing a post-translationally modification called poly(ADP-ribosyl)ation [17]. Upon PARP inhibition, cells accumulate unrepaired spontaneous SSBs, which are converted to DSBs during DNA replication owing to collapsed replication forks. PARP-inhibited cells rely on functional HR to repair these replication-associated DSBs in order to prevent the accumulation of unrepaired lethal DSBs [17], [18], [19]. Inhibition of PARP activity was shown to be highly cytotoxic to cancer cells with dysfunctional HR due to *BRCA1/2* deficiencies [20], [21]. Therefore, many PARP inhibitors have been developed and FDA-approved as anti-tumour molecules for various cancers, such as ovarian cancer, with either somatic or germline *BRCA1/2* mutations [22], [23]. However, HR-deficiency is highly unlikely to be limited to *BRCA* mutations, underscoring the importance of identifying additional HR components which may be utilized as therapeutic targets or as predictive biomarkers for patient stratification [19], [23], [24], [25], [26].

The family of MOBs (monopolar spindle-one-binder proteins) is highly conserved in eukaryotes [27], [28], [29], [30]. hMOB1 protein interacts with all four human STK38/LATS kinases, whereas hMOB2 forms a complex with STK38/STK38L but not with LATS1/2 kinases [31], [32], [33] and is shown to compete with hMOB1 for STK38 binding [34]. The hMOB1/STK38 complex is associated with increased STK38 activity, while hMOB2 binding to STK38 blocks kinase activation [34]. STK38/STK38L kinases can participate in the regulation of G1/S cell cycle progression [35], [36], apoptosis [37], [38], [39], autophagy [40] and DDR [41], [42]. Genome-wide screening for novel regulators of the DDR classified hMOB2 (also termed HCCA2) as a potential candidate [43]. Recent reports showed that hMOB2 supports cell survival upon extrinsic DNA damage induction [43], [44], [45]. Furthermore, the human *MOB2* gene appears to display loss-of-heterozygosity in more than 50% of bladder, cervical and ovarian carcinomas (TCGA) [46]. Our previous studies demonstrated that hMOB2 deficiency causes the accumulation of DNA damage, which in turn, activates the DDR kinases ATM and CHK2, consequently inducing a p53/p21-dependent G1/S checkpoint activation in the absence of any externally applied DNA damage [47]. We further found that hMOB2 interacts with RAD50, a component of the DNA damage sensor complex MRN (MRE11-RAD50-NBS1) and promotes recruitment of activated MRN and ATM to sites of damaged DNA [47]. hMOB2 also supports cancer cell survival and G1/S cell cycle arrest in response to exogenously induced DNA damage [47]. However, the full extent of the involvement of hMOB2 in the DDR at the molecular level has yet to be fully elucidated. In particular, it is unknown which type of DNA damage repair is supported by hMOB2.

Here we found that loss of hMOB2 disrupts HR-dependent DSB repair by interfering with the activation of RAD51, and the subsequent formation of RAD51 nucleofilaments on damaged DNA fragments. hMOB2 supports cancer cell survival in response to the DNA damaging compound bleomycin, and the DNA interstrand cross-linking (ICL) agents mitomycin C and cisplatin, all of which predominantly induce DSBs that require HR to be repaired. Most importantly, hMOB2 deficiency sensitise cancer cells to the PARP inhibitors olaparib, rucaparib and veliparib, revealing the possible therapeutic potential of PARP inhibitors for hMOB2-defective cancers. Taken together, our findings suggest that hMOB2 expression may represent a candidate biomarker when evaluating the suitability of targeted therapies for the treatment of HR defective cancers.

## 2 Materials and methods

### 2.1 Cell culture, transfections, and cell treatments

U2OS, HCT116, RPE1-hTert and PT67 cell lines were maintained in DMEM supplemented with 10% foetal calf serum (FCS). HOC7, OVCA 429, HEY, SKOV 3, OVCAR 3, OVCAR 8, IGROV 1 and OVCA 433 cell lines were maintained in DMEM supplemented with 10% FCS. U2OS DRGFP and EJ5GFP cells were maintained in DMEM without sodium pyruvate supplemented with 10% FCS [48]. Exponentially growing cells were transfected with siRNAs (Qiagen, sequences available upon request) using Lipofectamine RNAiMax (Invitrogen) according to the manufacturer's instructions. Plasmids were transfected using Fugene 6 (Promega) or Lipofectamine 2000 (Invitrogen) according to the manufacturer's instructions. Bleomycin (MedChemExpress), mitomycin C (Sigma), cisplatin (Sigma), NU-7441 (Selleckchem), olaparib (AZD-2281, Enzo/Axxora), rucaparib (AG-014699, Selleckchem), veliparib (ABT-888, Selleckchem) and KU-55933 (Calbiochem/Merck) were added as indicated.

### 2.2 Generation of stable cell lines and IR treatments of cells

Retroviral pools using pLXSN and pSuper.retro.puro plasmids were generated using PT67 retrovirus packaging cells and Lipofectamine 2000 transfection reagent (Invitrogen) as reported [47]. Irradiation with the indicated doses was performed at a rate of 5 Gy/min (215 kV, 12.0 mA, 1.0 mm Al filter) using an AGO HS 320/250 X-ray machine (AGO X-ray Ltd.) equipped with a NDI-321 stationary anode X-ray tube (Varian), and then processed for clonogenic, immunofluorescence or comet assays as described below.

### 2.3 Cell proliferation analysis

Cell proliferation assays were performed using kinetic live cell imaging system (The INCUCYTE™ Kinetic Imaging System, Essen BioScience). Confluency was automatically measured every two hours for the indicated times. The final analysis was carried out by the IncuCyte software (INCUCYTE™, 2011 Essen BioScience Inc., 2011A Rev2).

### 2.4 Immunoblotting and densitometry analysis

Immunoblotting was performed as described [31], [47]. Rabbit monoclonal anti-hMOB2 antibodies were produced in collaboration with Epitomics. The following antibodies were used in immunoblotting: ATM (Millipore, 07-1286, 1/1000), p-ATM Ser1981 (Santa Cruz, sc-47,739, 1/200), BRCA2 (Calbiochem, OP95, 1/500), p-BRCA2 Ser3291 [49], FANCD2 (Abcam, ab108928, 1/500), RAD51 (Santa Cruz, sc-8349, 1/1000), p-RAD51 Ser14 [50], RPA70 (Millipore, NA13, 1/100), RPA32/34 (Millipore, NA18, 1/1000), p-RPA32/34 Ser4/8 (Bethyl Labs, A300-245A, 1/1000), NBS1 (BD Biosciences, 611,870, 1/1000), p-NBS1 Ser343 (Cell Signalling, 3001, 1/500), p53 (Santa Cruz, sc-126, 1/1000), γH2AX (Cell Signalling, 9718, 1/500), HA (Cell Signalling, 3724, 1/1000) and GAPDH (Santa Cruz, sc-32,233, 1/1000). Polyclonal rat anti-tubulin (YL1/2) was produced in our laboratory. Densitometric analysis of immunoblots were performed using the ImageJ software (NIH).

### 2.5 Immunofluorescence

Cells were processed for immunofluorescence as described [31], [51]. Briefly, cells cultured on glass coverslips were fixed in 3%-paraformaldehyde/2%-sucrose solution for 15 min at room temperature, permeabilized for 2 min with 0.5% (vol/vol) Triton X-100 in PBS, blocked (10% goat serum in PBS) and incubated with primary antibodies overnight at 4C. The next day, following incubation with secondary antibodies and DAPI (Sigma) or Hoechst (Invitrogen, H3570) for 2 h, coverslips were mounted using Vectashield mounting medium (Vector Lab). Primary and secondary antibodies were used in immunofluorescence as follows: RAD51 (Santa Cruz, sc-8349, 1/50) (Abcam, ab63801, 1/1000), RPA70 (Millipore, NA13, 1/100), γH2AX (Cell Signalling, 9718, 1/50), cyclin-A (Santa Cruz, sc-271,682, 1/100) and Mitosin/CENPF (Abcam, Ab5, 1/200), anti-rabbit FITC (Stratech – Jackson, 711-095-152, 1/100), anti-mouse FITC (Stratech – Jackson, 715-095-151, 1/100), anti-rabbit Texas Red (Stratech – Jackson, 711-075-152, 1/100) and anti-mouse Texas Red (Stratech – Jackson, 715-075-151, 1/100). Images were acquired with an ApoTome fluorescence microscope (Zeiss) with a 40× objective lens and processed with AxioVision AxioVS40 V4.8.1.0 (Zeiss) and Photoshop CS5 (Adobe Systems Inc.).

### 2.6 Alkaline comet assay

Alkaline comet assay protocol was performed as previously described [52], [53]. Briefly, after one-hour treatment with the ICL-inducing agent cisplatin (100 μM), cells were harvested and frozen at indicated time points. Prior to analysis, cells were thawed, resuspended in ice cold media, irradiated (17.5Gy) while kept on ice in order to introduce a fixed number of spontaneous DNA strand breaks immediately prior to analysis. After lysis, electrophoresis and staining, individual cells were visualized using an inverted microscope (Nikon) and analysed using Komet Analysis software 4.02 (Andor Technology). Per sample/time point/experiment at least 50 cells were randomly selected from duplicate slides and individual DNA damage levels (ICL) were determined. Results were stated as percentage decrease in tail moment compared to untreated irradiated controls that is calculated as follows: % decrease in tail moment = (1 - (TMdi-TMcu/TMci-TMcu)) x 100, where: TMdi = tail moment of drug-treated irradiated sample, TMcu = tail moment of control, unirradiated, untreated, TMci = tail moment of control, irradiated, untreated. A greater decrease corresponds to a higher level of ICL. A non-irradiated drug-treated sample was included to account for drug-induced single-strand break damage (not observed).

### 2.7 Clonogenic survival assays

Clonogenic assays were performed as described [47]. Briefly, cells were seeded at predetermined densities in 6-well or 6-cm plates and allowed to adhere for 24 h, before being irradiated or drug treated as indicated, followed by three media washes. Cells were replenished with fresh complete medium every 3 days until colony size reached more than 50 cells per colony. Cells were then fixed with MeOH/acidic acid (3:1) solution for 5 min, followed by staining with 0.5% crystal violet (Sigma) for 15 min. The surviving fraction was calculated using the plating efficiencies of the corresponding non-treated controls as reference [54].

### 2.8 GFP-based DNA repair reporter assays

To examine the efficacy of HR and NHEJ pathways, we conducted DRGFP and EJ5GFP GFP-reporter assays as described [48]. In principle, DSBs are induced by transient expression of I-*Sce*I enzyme in the corresponding cells. The induced DSBs are then repaired by the relevant repair pathway, which generates a functional full-length GFP gene. The produced GFP expression levels are considered as a precise readout for the efficiency of repair pathway tested. Briefly, DRGFP U2OS or EJGFP U2OS cells were transiently transfected with siMOB2 or control (Qiagen) using Lipofectamine RNAiMAX (Invitrogen) according to the specifications. At 24 h post-transfection, the cells were transfected with an I-*Sce*I expression plasmid (pCBA-Scel) or empty vector (pCAGGS) using Fugene 6 (Promega). 72 h later, the GFP+ cells were analysed by a flow cytometer (BD LSRFORTESSA X-20). Cells transfected with pCBA-Sce and treated with either 10 μM ATM inhibitor for 60 h or 10 μM DNA-PK inhibitor for 60 h were used as assay controls for DRGFP (HR) or EJ5GFP (NHEJ), respectively. HA-tagged I-*Sce*I expression was monitored by immunoblotting using an anti-HA antibody.

### 2.9 Statistical analysis

Graphics and statistical analyses were carried out using the GraphPad Prism software. Data are presented as mean ± S.E.M., unless stated otherwise. Statistical significance was assessed applying one-tailed unpaired Student's *t*-test unless stated otherwise. For all tests, differences were considered statistically significant when *p*-values were below 0.05 (*), 0.01 (**), or 0.001 (***), respectively. p-values are indicated in the corresponding figure legends.

## 3 Results

### 3.1 hMOB2 supports DSB repair by homologous recombination

**Fig. 1:**
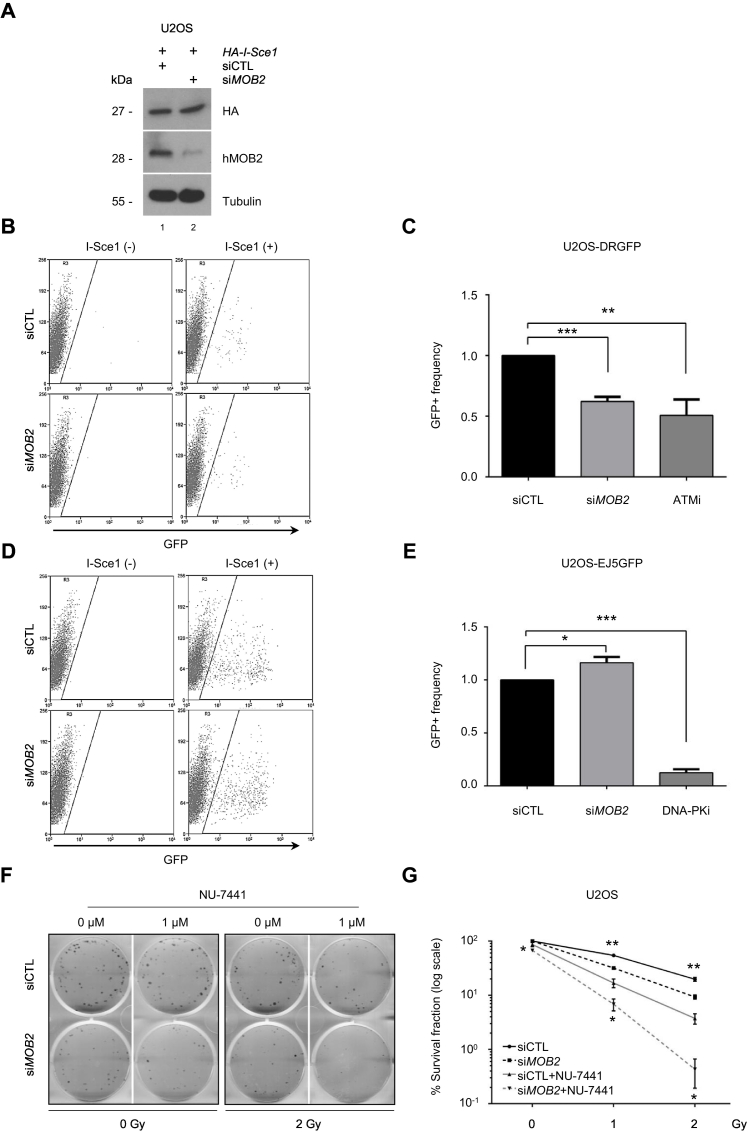
hMOB2 promotes HR to repair DNA double strand breaks. (A) Western blot showing expression of HA and hMOB2 in control or hMOB2-depleted U2OS DRGFP cells following transfection with empty vector or pCBA-Scel. (B) Representative GFP+ flow cytometry dot plots of U2OS DRGFP cells following transfection with empty vector or pCBA-Scel. (C) Quantification of the experimental groups shown in B. Cells treated with ATM inhibitor (KU-55933, 10 μM, 60 h) were used as positive control. Results display the relative frequency of GFP+ cells normalized to control cells, calculated according to raw GFP%, and are shown as mean ± S.E.M (n = 4, p- values: siMOB2 = 2.9E−04, ATMi = 9.7E−03 compared to siRNA control). (D) Representative GFP+ flow cytometry dot plots of U2OS EJ5GFP cells following transfection with empty vector or pCBA-Scel. (E) Quantification of the experimental groups shown in D. Cells treated with DNA-PK inhibitor (NU-7441, 10 μM, 60h) were used as positive control. Results display the relative frequency of GFP+ cells normalised to control cells, calculated according to raw GFP%, and are shown as mean ± S.E.M (n = 3, p-values: siMOB2 = 0.048, DNA-PKi = 6.8E−04 compared to siRNA control). (F) Representative images of the clonogenic survival assays in control and hMOB2-depleted U2OS cells treated with DNA-PK inhibitor (NU-7441, 10 μM, 1 h), combined with ionising radiation (IR) with the indicated doses and followed by NU-7441 treatment (10 μM, 16 h). (G) Quantification of the experimental groups shown in F. Results display the percentage (log scale) of colonies formed, corrected according to plating efficiencies of the corresponding untreated controls and are shown as mean ± S.E.M. (n = 3, p-values: 0 Gy: 0.021; 1 Gy, MOB2 = 0.004, MOB2/DNA-PKi = 0.029; 2 Gy, MOB2 = 0.005, MOB2/DNA-PKi = 0.022).

Loss of hMOB2 function induces unrepaired DSB accumulation, triggering DNA damage-dependent p53 signalling, which elevates p21 expression to arrest cells at the G1/S cell cycle checkpoint [47]. To understand the mechanistic reason for the spontaneous DSB accumulation upon hMOB2 depletion, HR and NHEJ functions were evaluated with direct repeat-GFP (DRGFP) reporter assays [48], [55]. First, HR was assessed using the DRGFP system [48], which allows the HR-directed repair of DSBs induced by the I-*Sce*I endonuclease in a mutated GFP gene (Supplementary Fig. S1A). Upon I-SceI expression, hMOB2-depleted cells (Fig. 1A) showed nearly 50% decrease in HR activity (Fig. 1B and C), when compared to control cells. The ATM inhibitor KU-55933, previously reported to prevent HR in this DRGFP system [48], acted as the positive control. Cells with dysfunctional HR rely on NHEJ to resolve DSBs [1], [6], hence the EJ5GFP assay (Supplementary Fig. S1B) was used to investigate NHEJ activity [48]. Not only was NHEJ not reduced in hMOB2-defective cells, it even displayed a slight increase in activity (Fig. 1D and E), suggesting a compensatory effect in hMOB2-depleted cells. The DNA-PK inhibitor NU-7441 was used as a positive control for the NHEJ assay [56]. Given that hMOB2-deficient cells exhibited significantly attenuated HR and increased NHEJ to repair DSBs, we next investigated whether inhibition of NHEJ may display synthetic lethality in combination with hMOB2 knockdown, with or without exogenous DNA damage. NU-7741 treatment decreased the survival of hMOB2-deficient cells in the absence or presence of DSBs caused by IR-treatment (Fig. 1F and G). We did not observe a radio-sensitising effect of hMOB2 downregulation in ATM-inhibited cells, which presumably is a consequence of both hMOB2 and ATM acting in the same DDR pathway (Supplementary Fig. S1C). Together, these findings suggest that the inhibition of hMOB2- and DNA-PK-dependent pathways can display cytotoxic effect, especially upon exposure to radiation, which is likely a result of elevated dependency of NHEJ in hMOB2-depleted cells.

### 3.2 RAD51 loading is defective in hMOB2-deficient cells

**Fig. 2:**
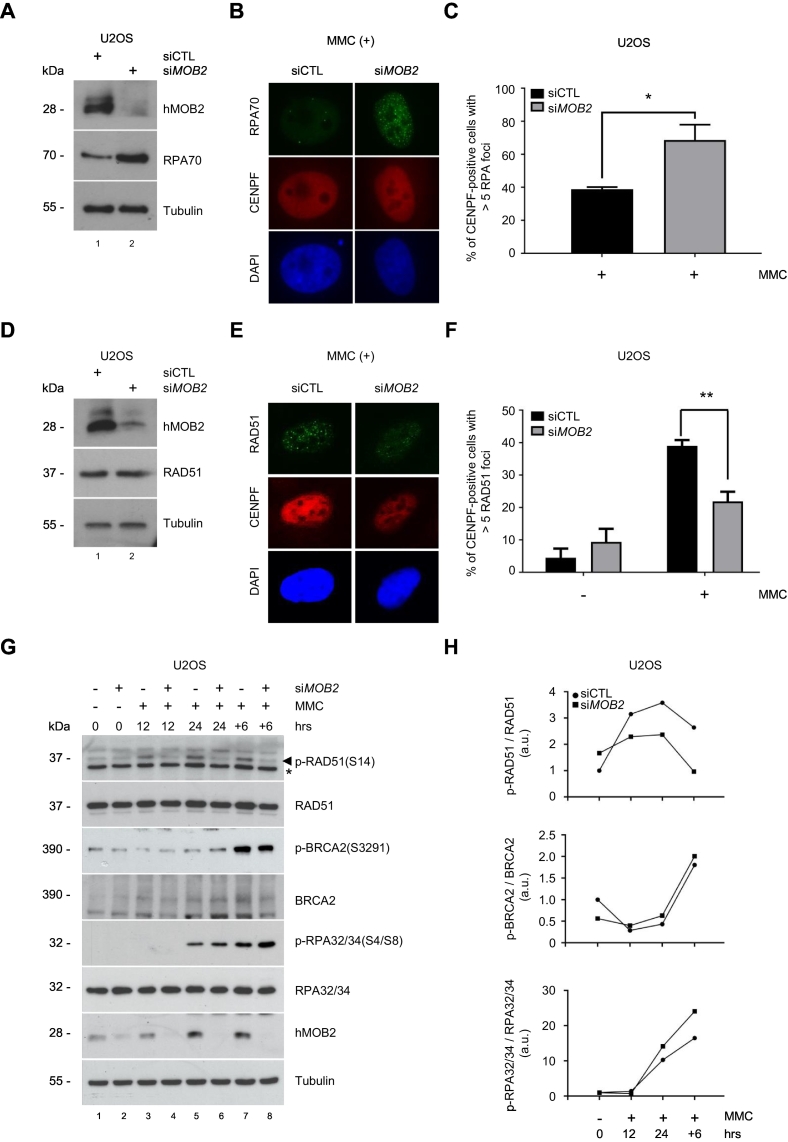
hMOB2 supports RAD51 phosphorylation and RAD51 recruitment onto damaged DNA. (A) Western blot showing expression of hMOB2 and RPA70 in control or hMOB2-depleted U2OS cells. (B) Representative images of RPA70 (green) and CENPF (red) in control or hMOB2-depleted U2OS cells following mitomycin C treatment (MMC, 0.3 μM). DAPI (blue) is used to stain cell nuclei. (C) Quantification of B. Results displaying percentages of mitomycin C-treated CENPF-positive U2OS cells with more than five RPA70 foci are shown as mean ± S.E.M. (n = 3, >150 cells/experiment; p = 0.041). (D) Western blot showing expression of hMOB2 and RAD51 in control or hMOB2-depleted U2OS cells. (E) Representative images of RAD51 (green) and CENPF (red) in control or hMOB2-depleted U2OS cells following mitomycin C treatment (MMC, 0.3 μM). DAPI (blue) is used to stain cell nuclei. (F) Quantification of E. Results displaying nuclear RAD51 foci number in CENPF-positive U2OS cells treated with or without mitomycin C are shown as mean ± S.E.M. (n = 3, >150 cells were scored per experiment; p < 0.0001, Mann– Whitney test). (G) Western blot showing phosphorylation of RAD51, BRCA2 and RPA32/34 in control or hMOB2-depleted U2OS cells following mitomycin C treatment (MMC, 0.3 μM, indicated timepoints, +6: further incubation in drug-free medium for 6 h). Asterisks (*) mark an unspecific band in the RAD51 blots. (H) Quantification of G. Results showing densitometry quantification of Western blots shown in G (phosphorylated/total protein).

The early HR response mechanism comprises three essential steps; 3′ single-strand DNA (ssDNA) generation by 5′- 3′ end resection, recruitment of RPA on resected 3’ ssDNA overhangs, and displacement of RPA by RAD51 assisted by the BRCA1-PALB2-BRCA2 complex to form RAD51 nucleoprotein filaments [1], [8], [57]. Therefore, we next sought to determine whether the inefficient HR in hMOB2-depleted cells was a consequence of defective ssDNA formation, using RPA70 foci formation as a read-out for ssDNA formation. To rule out any cell cycle-specific effects, only S/G2 cells (i.e., only those cells capable of performing HR) that were positive for the centromere protein F (CENPF) were selected. In cells treated with the DNA crosslinking agent mitomycin C, hMOB2 depletion revealed a two-fold increase in the number of CENPF-positive cells with more than five RPA foci when compared to control cells (Fig. 2A, B and C). Loss of hMOB2 also caused augmented RPA protein levels compared to controls (Fig. 2A). Impaired formation of RPA was restored by exogenous expression of RNAi-resistant hMOB2 in hMOB2-depleted cells (Supplementary Fig. S2A–C). Thus, ssDNA formation does not appear to be impaired in hMOB2-deficient cells, rather elevated levels of ssDNA formation seems to occur.

Next, we tested whether dysfunctional HR activity was a consequence of compromised RAD51 nucleofilament formation upon hMOB2 knockdown in U2OS cells. hMOB2-depleted (Fig. 2D) CENPF-positive cells treated with mitomycin C displayed a decrease in RAD51 nucleofilament formation (Fig. 2E and F), without affecting total RAD51 protein levels (Fig. 2D). We further analysed formation of RAD51 ionising radiation (IR)–induced foci (IRIF) in cyclin A–positive cells, confirming that hMOB2-deficient U2OS cells displayed a significant reduction in RAD51 formation (Supplementary Fig. S2D and E). DNA damage induction was similar in both conditions, as judged by γ-H2AX phosphorylation at S139 (Supplementary Fig. S2F) and γ-H2AX foci formation (Supplementary Fig. S2G and H). Thus, our results propose that hMOB2 deficiency can result in impaired replacement of RPA by RAD51 polymers to form nucleoprotein filaments, further supporting our previous observation of compromised HR function upon hMOB2 deficiency.

Consequently, we investigated next the mechanism underlying impaired RAD51 loading onto damaged DNA in hMOB2-silenced U2OS cells. In response to DNA damage or during the routine progression of the cell cycle, reduced CDK-dependent Ser3291 phosphorylation of BRCA2 was reported to promote interaction between the BRCA2 C-terminus and RAD51, which consequently stimulates and stabilizes RAD51 nucleofilament formation on ssDNA [49], [58], [59]. Furthermore, RAD51 phosphorylation by the PLK1 and CK2 kinases is essential for efficient RAD51 loading mediated by BRCA2 [50], [60], [61], [62]. Additionally, DNA-PK-dependent RPA32/34 phosphorylation at Ser4/Ser8 was reported to suppress unscheduled HR activity during the cell cycle [63]. Therefore, these three types of regulation by phosphorylation were examined (Fig. 2G and H). Regulatory phosphorylations of BRCA2 at Ser3291 and RPA32/34 at Ser4/Ser8 were not reduced upon hMOB2 depletion (Fig. 2G and H). In stark contrast, Ser14 phosphorylation of RAD51 was decreased in hMOB2-deficient cells, as early as 12 h after the induction of DNA damage. This impairment was sustained after removal of the genotoxic insult (+6 h, Fig. 2G and H). Collectively, as depicted in Supp. Figure-5, our data would suggest that, upon hMOB2 deficiency, the regulatory phosphorylation of RAD51 is impaired, resulting in decreased RAD51 loading onto ssDNAs, finally causing an impairment of HR activity as a result of defective RAD51 activation in spite of the formation of RPA-loaded ssDNA.

### 3.3 hMOB2 loss sensitises cancer cells to DSB-inducing drugs

**Fig. 3:**
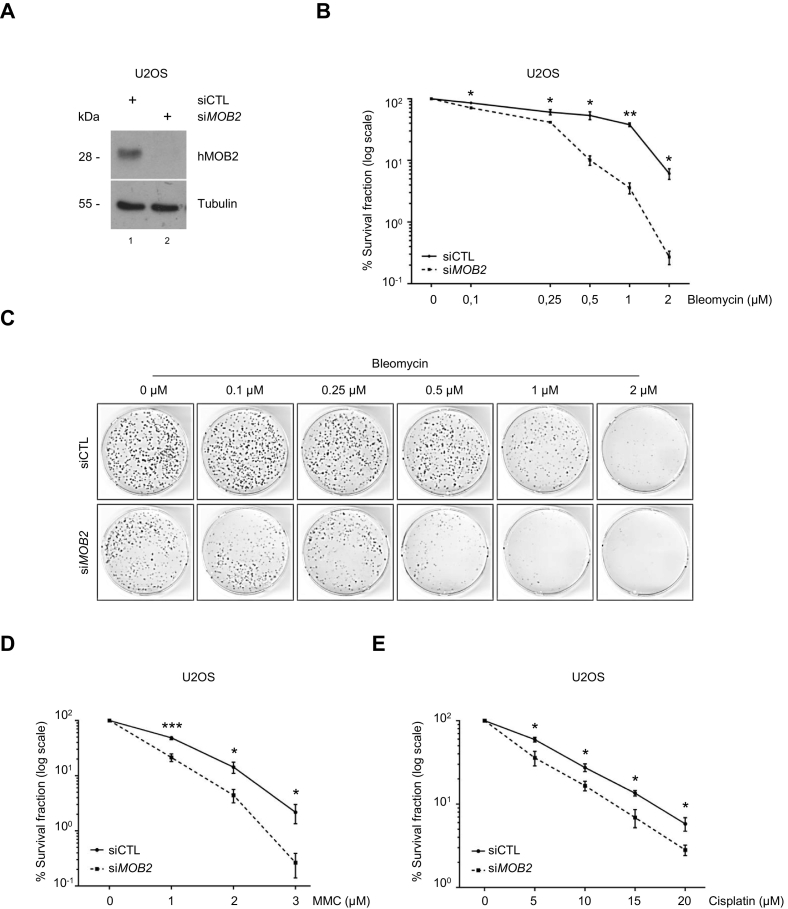
hMOB2 supports cancer cell survival in response to DSB-inducing anti-cancer agents. (A) Western blot showing expression of hMOB2 in control or hMOB2-depleted U2OS cells. (B) Survival of control or hMOB2-depleted U2OS cells following bleomycin treatment (72 h, indicated doses). Results showing the percentage (log scale) of colonies formed after treatment with indicated doses, corrected according to the plating efficiencies of the corresponding untreated controls, and shown as mean ± S.E.M. (*n* = 4, p-values: 1 μM = 8.3E−04, 2 μM = 0.025, 3 μM = 0.021). (C) Representative images of the clonogenic survival assays shown in B. (D) Survival of control or hMOB2-depleted U2OS cells following mitomycin C treatment (MMC, 1 h). Results showing the percentage (log scale) of colonies formed after treatment with indicated doses, corrected according to the plating efficiencies of the corresponding untreated controls, and shown as mean ± S.E.M. (n = 4, p-values: 1 μM = 8.3E-04, 2 μM = 0.025, 3 μM = 0.021). (E) Survival of control or hMOB2-depleted U2OS cells following cisplatin treatment (1 h). Results showing the percentage (log scale) of colonies formed after treatment with indicated doses, corrected according to the plating efficiencies of the corresponding untreated controls, and shown as mean ± S.E.M. (n = 4, p-values: 5 μM = 0.018, 10 μM = 0.013, 15 μM = 0.01, 20 μM = 0.031).

We have previously shown that loss of hMOB2 can sensitise cancer cells to IR-treatment [47], which mainly exerts damage on DNA by producing catastrophic DSBs [64]. Other chemotherapeutics including the DNA-damaging antibiotic bleomycin [65], and the DNA interstrand crosslinkers mitomycin C and cisplatin [66], [67] can disrupt replication, thus producing replication-associated DSBs whose repair mainly relies on HR [7], [64], [68], [69], [70]. Thus, we conducted survival assays to test whether hMOB2 silencing renders cancer cells sensitive to these replication stress-inducers. hMOB2 knockdown (Fig. 3A) potentiated the cytotoxicity of bleomycin (Fig. 3B and C), mitomycin C (Fig. 3D) and cisplatin (Fig. 3E) treatments. Notably, hMOB2 protein levels were significantly increased upon bleomycin treatment, suggesting a potential requirement for hMOB2 in the response to bleomycin-induced DNA damage (Supplementary Fig. S3A). The modified alkaline comet assay did not show differences in ICL induction or repair kinetics of mitomycin C or cisplatin between hMOB2-proficient and -deficient cells, ruling out inconsistent ICL adduct formation being responsible for the increased cytotoxicity of ICL agents in hMOB2-depleted cells (Supplementary Fig. S3B and C). To directly establish that the involvement of hMOB2 in HR repair was independent of the Fanconi Anemia (FA) pathway-coordinated ICL unhooking process, we transiently co-depleted hMOB2 and FANCD2 (Supplementary Fig. S3D), a central component of the FA pathway [66], [67], and subsequently analysed sensitivity to mitomycin C. Cells with co-depletion of hMOB2 and FANCD2 exhibited potentiated cytotoxicity to mitomycin C treatment, when compared with single depletions (Supplementary Fig. S3E), suggesting a role for hMOB2 in ICL repair that is independent of the canonical FA pathway. Taken together, these results support our notion that hMOB2 depletion impairs HR in cells suffering from excessive ICLs, consequently resulting in cancer cells becoming sensitive towards anti-cancer agents that can induce DSBs.

### 3.4 hMOB2 loss enhances the anti-tumour activity of PARP inhibitors

**Fig. 4:**
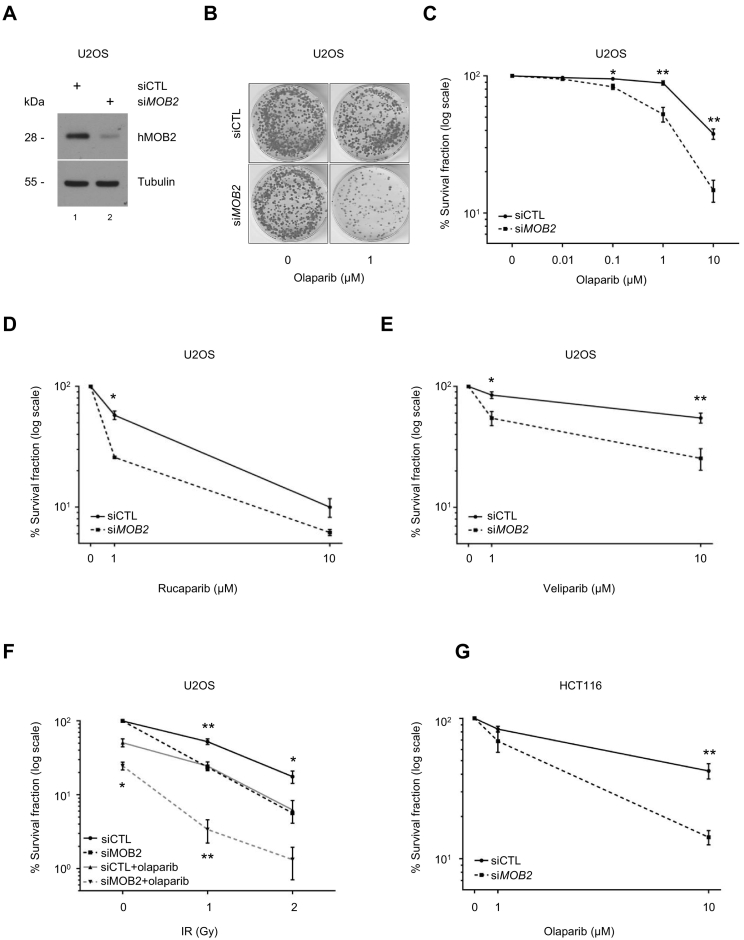
hMOB2 deficiency renders cancer cells vulnerable to PARP inhibition. (A) Western blot showing expression of hMOB2 in control or hMOB2-depleted U2OS cells. (B) Representative images of the clonogenic survival assays in control or hMOB2-depleted U2OS cells. (C) Survival of control or hMOB2-depleted U2OS cells following olaparib treatment (AZD-2281; 0.01, 0.1, 1 μM or 10 μM; 24 h). Results showing the percentage (log scale) of colonies formed after treatment with indicated doses, corrected according to the plating efficiencies of the corresponding untreated controls, and shown as mean ± S.E.M. (*n* = 3, p-values: 0.1 μM = 0.029, 1 μM = 0.008, 10 μM = 0.003). (D) Survival of control or hMOB2-depleted U2OS cells following rucaparib treatment (AG-014699, 1 μM or 10 μM, 24 h). Results showing the percentage (log scale) of colonies formed after treatment with indicated doses, corrected according to the plating efficiencies of the corresponding untreated controls, and shown as mean ± S.E.M. (n = 3, p-values: 1 μM = 0.01). (E) Survival of control or hMOB2-depleted U2OS cells following veliparib treatment (ABT-888, 1 μM or 10 μM, 24 h). Results showing the percentage (log scale) of colonies formed after treatment with indicated doses, corrected according to the plating efficiencies of the corresponding untreated controls, and shown as mean ± S.E.M. (n = 3, p- values: 1 μM = 0.017, 10 μM = 0.008). (F) Survival of control or hMOB2-depleted U2OS cells following olaparib treatment (AZD-2281, 1 μM, 1 h) in combination with indicated doses of IR followed by a 23 h control or olaparib treatment (AZD-2281, 1 μM). Results showing the percentage (log scale) of colonies formed after treatment with indicated doses, corrected according to the plating efficiencies of the corresponding untreated controls, and shown as mean ± S.E.M. (n = 3, p-values: 0Gy, MOB2/PARPi = 0.017; 1 Gy, MOB2 = 0.007, MOB2/PARPi = 0.007; 2 Gy, MOB2 = 0.035). (G) Survival of control or hMOB2-depleted HCT116 cells following olaparib treatment (AZD-2281, 1 μM or 10 μM, 24 h). Results showing the percentage (log scale) of colonies formed after treatment with indicated doses, corrected according to the plating efficiencies of the corresponding untreated controls, and shown as mean ± S.E.M. (n = 4, p-values: 10 μM = 0.004).

Given that several lines of evidence strongly suggest that cancer cells with HR deficiency (e.g. through *BRCA1/2* inactivation) show a significant hypersensitivity to PARP inhibition [17], [19], [20], [21], [24], [26], and given that hMOB2 deficiency impairs HR in cancer cells (see Fig. 1, Fig. 2, Fig. 3), we next sought to determine whether hMOB2 loss has a synthetic lethal interaction with PARP inhibition. hMOB2-depleted cells (Fig. 4A) were subjected to PARP inhibition. hMOB2 deficiency sensitised cancer cells towards three different FDA-approved PARP inhibitors; olaparib (Fig. 4B and C), rucaparib (Fig. 4D) and veliparib (Fig. 4E). Furthermore, hMOB2 downregulation augments the radio-sensitising effect of olaparib treatment (Fig. 4F). hMOB2 knockdown further potentiates the cytotoxicity of olaparib combined with NU-7441 (Supplementary Fig. S4A). Additionally, hMOB2 rendered HCT116 colorectal cancer cells susceptible to olaparib treatment (Fig. 4G). The augmented cytotoxicity of PARP inhibitors in hMOB2-depleted cells was proliferation independent since hMOB2 knockdown did not interfere with in the proliferation of HCT116 cells (Supplementary Fig. S4B). Ectopic expression of RNAi-resistant hMOB2 in hMOB2-depleted cells (Supplementary Fig. S4C) confirmed the restoration of cell survival upon challenge with the ICL-inducing agent mitomycin C (Supplementary Fig. S4D and E) or the PARP inhibitor olaparib (Supplementary Fig. S4F). Collectively, these results suggest that hMOB2 supports cancer cell survival upon the inhibition of PARP enzymes.

**Fig. 5:**
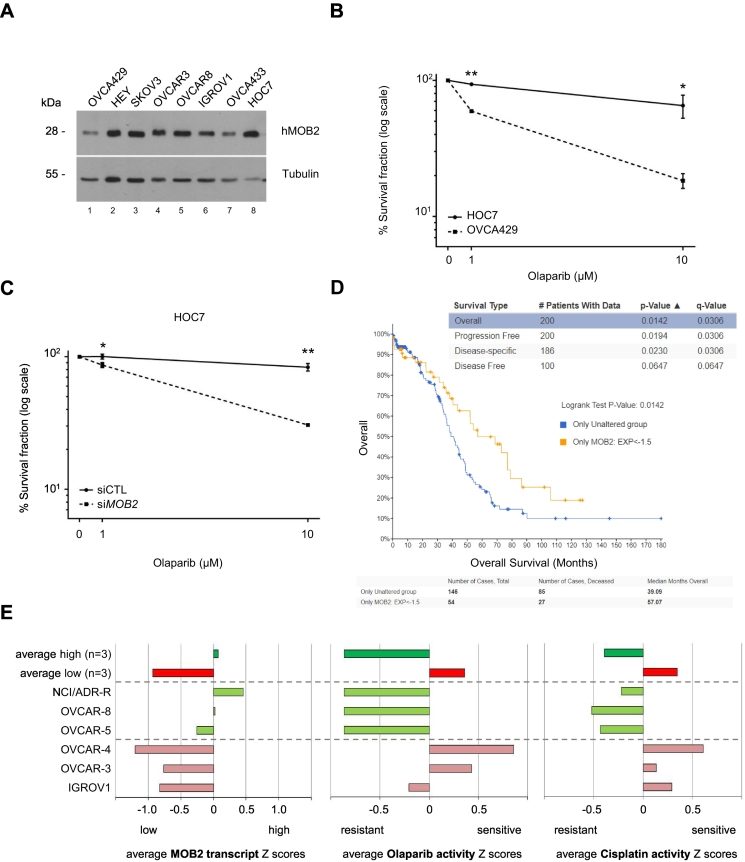
hMOB2 expression may serve as an indicator of responsiveness to PARP inhibition. (A) Western blot showing expression of hMOB2 in a panel of ovarian cell lines. (B) Survival of HOC7 and OVCA429 cells following olaparib treatment (AZD-2281, 1 μM or 10 μM, 24 h). Results showing the percentage (log scale) of colonies formed after treatment with indicated doses, corrected according to the plating efficiencies of the corresponding untreated controls, and shown as mean ± S.E.M. (n = 3, p-values: 1 μM = 1.3E-03, 10 μM = 0.03). (C) Survival of control or hMOB2-depleted HOC7 cells following olaparib treatment (AZD-2281, 1 μM or 10 μM, 24 h). Results showing the percentage (log scale) of colonies formed after treatment with indicated doses, corrected according to the plating efficiencies of the corresponding untreated controls, and shown as mean ± S.E.M. (n = 3, p-values: 1 μM = 0.04, 10 μM = 0.004). (D) Lower hMOB2 mRNA levels (orange line) correlate with increased survival of ovarian carcinoma patients. cBioPortal analysis of the TCGA OV data set (https://www.cbioportal.org/study/summary?id=ov_tcga_pan_can_atlas_2018) (E) MOB2 transcript levels inversely correlate with olaparib and cisplatin sensitivity in human ovarian cancer cells. Bar graphs representing the analysis of human ovarian cancer cell lines whose MOB2 transcript levels *(left panel)*, olaparib and cisplatin sensitivities (*middle and right panel respectively*) are publicly available on the CellMiner browser (https://discover.nci.nih.gov/cellminer/). The average values of the cell lines with high (green) and low (red) MOB2 mRNA levels are also included. Notably, low MOB2 mRNA levels correlate with increased sensitivities to olaparib and cisplatin treatments, further supporting our notion that endogenous MOB2 levels can correlate with olaparib sensitivities.

To further consolidate the functional significance of hMOB2 expression in the context of clinical responses to PARP inhibitor, we tested whether there is a correlation between hMOB2 expression and olaparib sensitivity. In that regard, the publicly available data suggests the ovarian cancer as an appropriate model to study this relevance. The levels of hMOB2 in a panel of human ovarian cancer cell lines were determined (Fig. 5A). OVCA429 and HOC7 cells displayed the lowest versus highest hMOB2 expression in this panel (Fig. 5A, lanes 1 and 8). Notably, both cell lines are *BRCA1/2* wild-type [71]; hence any difference between OVCA429 and HOC7 cells cannot be attributed to defective *BRCA1/2*. Higher olaparib sensitivity was associated with lower hMOB2 protein levels among these two *BRCA1/2* wild-type ovarian cancer cell lines (Fig. 5B). Moreover, the olaparib resistance of HOC7 cells was reversed following hMOB2 depletion (Fig. 5C), underlining the negative correlation between hMOB2 levels and olaparib sensitivity in cancer cells.

Analysis of the publicly available TCGA data set for ovarian cancer found that decreased hMOB2 mRNA levels are linked to increased overall survival of patients suffering from ovarian cancer (Fig. 5D). In addition, examination of the publicly available CellMiner database found that ovarian cancer lines with lower *MOB2* mRNA levels seem to be more sensitive to olaparib or cisplatin treatments when compared to cells expressing higher MOB2 mRNA levels (Fig. 5E). Taken together, these findings from databases further support our notion that hMOB2 level might hold the promise to serve as future marker in predicting the response to PARP inhibitors in patients.

## 4 Discussion

Cells need a fully functional DNA damage response (DDR) to avoid accumulation of genetic errors that can drive genomic instability and tumorigenesis [1]. An inefficient DDR and the persistence of unrepaired DNA damage leads to an accumulation of mutations that can induce cell death, senescence, or transformation [4], [72], [73]. However, those proteins responsible for DDR deficiencies can be exploited as predictive biomarkers or pharmacological targets [10], [11], [13], [14], [74], [75], and therefore, enable us to design more selective and specific cancer treatment strategies. In this regard, we describe here hMOB2 as a potential marker of interest that could be exploited in therapies targeting DDR deficiencies. Given that hMOB2 is an intracellular protein that does not display any enzymatic activity, it is unlikely that hMOB2 can be targeted by conventional drug discovery approaches. However, the advent of novel approaches targeting protein-protein interaction [76] and repurposing of E3 ligases for targeted degradation [77], [78] might allow hMOB2 targeting in the future, although much remains to be learned in upcoming studies to empower such drug discovery angles. In particular, it would be of interest to determine whether the link of hMOB2 to STK38/STK38L [79] could be exploited.

Previously, a genome-wide screen for novel DDR factors identified hMOB2 as a potential candidate [43] and we previously reported that hMOB2 depletion causes the accumulation of DSBs, which in turn activates DDR signalling to activate the p53/p21-dependent G1/S cell cycle checkpoint [47]. Therefore, we investigated how hMOB2 can support DSB repair. Our data would suggest that hMOB2 is required for an efficient repair of DSBs through HR, which helps to explain the cell cycle-dependent accumulation of DSBs upon hMOB2 loss [47]. NHEJ activity, however, is increased in hMOB2-depleted cells, and NHEJ inhibition by a DNA-PK inhibitor in hMOB2-depleted cells uncovered a synthetical lethal interaction. Thus, it is likely that in hMOB2-deficient cells NHEJ mediated DSB repair attempts to compensate for decreased HR mediated repair, which has already been described for deficiencies of other regulators of DSB repair [6], [8], [69].

In HR-mediated DSB repair, 5′ ends are subjected to exonucleolytic resection, which is initially performed by Mre11 and CtIP and further extended by Exo1 and DNA2 nucleases, generating 3′-ended single-stranded DNA (ssDNA) fragments. The ssDNA overhangs are rapidly coated by the heterotrimeric RPA complex, which is eventually replaced by RAD51 recombinase, consequently mediating DNA strand invasion and HR completion [5], [6], [7], [8], [62], [69]. We found that the initial steps of HR are functional in hMOB2-deficient cells, as evidenced by intact RPA recruitment. However, the displacement of RPA by RAD51 was impaired in hMOB2-depleted cells, helping to mechanistically explain why hMOB2-deficient cells accumulate DSB lesions.

In response to DNA damage, the PLK1 kinase phosphorylates RAD51 at serine 14, and this modification is required for proper RAD51 nucleofilament formation [50], [62]. In this regard, we discovered that hMOB2 deficiency impairs PLK1-dependent RAD51 phosphorylation, providing a molecular explanation for why hMOB2-deficient cells display compromised RAD51 loading despite abundant RPA loading. Now, future research is needed to dissect how hMOB2 supports PLK1-mediated RAD51 phosphorylation. In this regard, it is noteworthy that PLK1-dependent phosphorylation of STK38 can function as a switch to control hMOB1 vs. hMOB2-binding to STK38 in the context of spindle orientation during mitosis [80]. Thus, the STK38-hMOB2 axis might be linked to RAD51 regulation by PLK1. STK38/STK38L kinases have recently been linked to different aspects of the DDR [41], [81] although compensatory mechanisms were reported upon STK38 or STK38L loss-of-function, respectively [39], [82]. Thus, future research is warranted to examine whether and how PLK1-STK38-hMOB2 signalling may contribute to DSB repair through the regulation of RAD51.

In full agreement with a previous genome-wide DDR screen proposing that hMOB2 loss may contribute to mitomycin C sensitivity [43], we found that hMOB2 supports cancer cell survival in response to treatments with the radiomimetic drug bleomycin and the DNA crosslinking agents mitomycin C and cisplatin, with all 3 compounds being able to trigger the formation of DSBs [65], [66]. In this regard, one should note that cancer cells rely on a variety of compensatory DDR pathways to ensure their survival, in particular in response to potentially lethal DSBs [1], [70], [83], [84]. Over the past decade, a large amount of research has been conducted to decipher how these compensatory mechanisms could be exploited in the form of synthetic lethality, in order to achieve more selective and efficient cancer treatments [10], [14], [24], [26], [74], [75], [84]. PARP inhibition in HR-deficient cancer cells has emerged as a very promising prototype of exploitable synthetic lethality [20], [21]. Various PARP inhibitors have been FDA-approved to treat patients with *BRCA*-mutated breast, ovarian, pancreatic and prostate cancers [14], [23]. However, *BRCA1/2* mutations have relatively low frequency, which restricts the clinical usage of PARP inhibitors [25], [85]. Thus, dysfunctionalities of other DDR factors involved in HR-mediated DSB repair, such as RAD51, ATR, ATM, and CHK1/2, are being studied to expand the spectrum of cancer patients that could benefit from PARP inhibitor treatments [14], [26], [86], [87]. These studies underscore the importance of discovering HR-linked components that hold the potential to be utilized as therapeutic targets and/or as predictive markers for patient stratification with regard to PARP inhibition. Our data show that cancer cells with hMOB2 loss become more sensitive to the PARP inhibitors olaparib, rucaparib and veliparib. In particular, we found that ovarian cancer cells seem to represent a cancer type that is worthy to be explored further. Our findings together with publicly available cell line profiling data [88] would suggest that ovarian cancer cells display a link between MOB2 mRNA levels and sensitivity to PARP inhibition and a standard genotoxic therapeutic agent, such as cisplatin. In samples of patients suffering from ovarian cancer decreased hMOB2 levels are linked to improved patient survival, possibly due to elevated responsiveness to standard-of-care DNA damaging agents. Collectively, these findings would suggest that low hMOB2 levels may represent a promising biomarker for increased responsiveness to agents, such as PARP inhibitors, exploiting a potential synthetic lethal interaction associated with HR deficiency. Thus, future research is warranted to understand how patient stratification and treatment choice can be based on *MOB2* expression, in a similar fashion as reported recently for other DDR candidates [87].

## 5 Conclusion

The data presented herein reveal that hMOB2 deficiency interferes with PLK-mediated RAD51 phosphorylation, and therefore, RAD51 nucleofilament formation on damaged chromatin, which is an indispensable step in the HR-dependent resolution of DSBs. Since hMOB2 supports efficient HR-mediated DSB repair, loss of hMOB2 can render cancer cells sensitive to DSB-inducing DNA damaging agents and PARP inhibitors. Consequently, our study provides a concept based upon which hMOB2 expression should possibly be considered as a candidate biomarker in the evaluation for personalised cancer therapies involving PARP inhibitors and other agents that exploit synthetic lethality associated with HR deficiency.

## Author contributions

AH conceived the study. RG, VG and AH designed, analysed and interpreted the majority of experiments supported by JH and FE. RG, AH and VG wrote the manuscript. RG performed the majority of experiments supported by MKE, AD, VS, JJGG. All authors contributed with discussion and edited the manuscript.

## Declaration of competing interest

The authors declare that they have no conflict of interest.

## Appendix A Supplementary data

Supplementary material 1 Image 1

Supplementary material 2 Image 1
