# Supplementary material for: hMOB2 deficiency impairs homologous recombination-mediated DNA repair and sensitises cancer cells to PARP inhibitors
Source: Cell Signal. 2021 Nov;87:110106. doi: 10.1016/j.cellsig.2021.110106 (PMC8514680; doi:10.1016/j.cellsig.2021.110106)
Supplement: Supplementary file 1 — Supplementary material 1 [file mmc1.docx]

**Supplementary Figure Legends**

**Figure-S1**

**hMOB2 depletion does not synergise with ATM inhibition.**

**(A, B)** Schematic diagrams of the HR (DRGFP) or NHEJ (EJ5GFP) reporter assays, respectively. Adapted from Gunn and Stark (2012). iGFP: internal GFP, puro: puromycin. **(C)** Survival of control or hMOB2-depleted U2OS cells following ATM inhibitor treatment (KU-55933, 1 μM, 1 h) in combination with IR (2 Gy) followed by a 23 h control or ATM inhibitor treatment (KU-55933, 1 μM). Results showing the percentage (log scale) of colonies formed after treatment with indicated doses, corrected according to the plating efficiencies of the corresponding untreated controls, and shown as mean ± S.E.M. (n=3, p-values: 2 Gy, MOB2=0.003, MOB2/ATMi=0.006).

**Figure-S2**

**hMOB2 depletion does not impair γH2AX foci formation upon MMC treatment.**

**(A)** Western blot showing expression of RPA70, hMOB2 and HA in control or hMOB2-depleted U2OS cells stably expressing empty vector (EV) or wild-type HA-MOB2. **(B)** Representative images of RPA70 foci (green) and CENPF (red) in control or hMOB2-depleted U2OS cells stably expressing empty vector (EV) or wild-type HA-MOB2 upon 0.3 μM mitomycin C. DAPI (blue) is used to stain cell nuclei. **(C)** Quantification of B. Results displaying percentages of mitomycin C-treated CENPF-positive U2OS cells with more than five RPA70 foci are shown as mean ± S.E.M. (n=3, >150 cells/experiment). MMC: Mitomycin C. **(D)** Representative images of RAD51 foci (green) and cyclin-A (red) in control or hMOB2-depleted U2OS cells treated with 2 Gy IR treatment. DAPI (blue) is used to stain cell nuclei. **(E)** Quantification of D. Results displaying percentages of IR-treated or untreated CENPF-positive U2OS cells with more than five RAD51 foci are shown as mean ± S.E.M. (n=3, >150 cells/experiment). IR: ionising radiation. **(F)** Western blot showing expression of hMOB2 and γH2AX in control or hMOB2-depleted U2OS cells. **(G)** Representative images of γH2AX foci (red) and CENPF (green) in control or hMOB2-depleted U2OS cells treated with 0.3 μM mitomycin C. DAPI (blue) is used to stain cell nuclei. **(H)** Quantification of B. Results displaying percentages of mitomycin C-treated or untreated CENPF-positive U2OS cells with more than five γH2AX foci are shown as mean ± S.E.M. (n=3, >150 cells/experiment). MMC: Mitomycin C.

**Figure-S3**

**hMOB2 protein levels increase upon DNA damage and augmented ICL-sensitivity of hMOB2-depleted cells is likely not caused by inconsistent formation of ICL adducts.**

**(A)** Western blot showing expression of hMOB2 following a 24h bleomycin treatment with the indicated doses. **(B)** Western blot showing expression of hMOB2 in control or hMOB2-depleted U2OS cells. **(C)** Quantification of comet assay displaying induced interstrand crosslink (ICL) formation in control or hMOB2-depleted U2OS cells following cisplatin treatment (100 μM, 1 h) and a recovery period in drug-free media for the indicated time points. Results are expressed as percentage decrease in tail moment mean and shown as mean ± S.E.M. (n=3, 50 cells/experiment, p-values: 0 h=0.31, 6 h=0.325, 9 h=0.473, 12 h=0.1, 48 h=0.067). **(D)** Western blot showing expression of hMOB2 and FANCD2 in control, FANCD2-depleted and/or hMOB2-depleted U2OS cells. **(E)** Survival of control, FANCD2-depleted and/or hMOB2-depleted U2OS cells following mitomycin C treatment (0.3 μM, 1 h). Results showing the percentage (log scale) of colonies formed after treatment with indicated doses, corrected according to the plating efficiencies of the corresponding untreated controls, and shown as mean ± S.E.M. (n=3, p-values: 0 μM, CTL- MOB2/FANCD2=0.003; 2 μM, CTL-MOB2=0.04, CTL-FANCD2 =0.012, CTL- MOB2/FANCD2=0.011, MOB2-MOB2/FANCD2=5.7E-03, FANCD2- MOB2/FANCD2=0.048).

**Figure-S4**

**hMOB2 supports the survival of U2OS and HCT116 cancer cells upon PARP inhibition.**

**(A)** Survival of control or hMOB2-depleted U2OS cells following olaparib treatment (NU-7441, 1 μM) in combination with olaparib (AZD-2281, 1 μM) for 24 h. Results showing the percentage (log scale) of colonies formed after treatment with indicated doses, corrected according to the plating efficiencies of the corresponding untreated controls, and shown as mean ± S.E.M. (n=3, p-values: 0 μM, MOB2/DNA-PKi=0.036; 1 μM, MOB2=0.003, MOB2/DNA-PKi=0.003). **(B)** Confluency percentages for the indicated timepoints in control or hMOB2-depleted HCT116 cells (n=3). **(C)** Western blot showing expression of hMOB2 and HA in control or hMOB2-depleted U2OS cells stably expressing empty vector (EV) or wild-type HA-MOB2. **(D)** Survival of control or hMOB2-depleted U2OS cells stably expressing empty vector (EV) or HA- MOB2 following mitomycin C treatment (2 μM, 24h). **(E)** Results showing the percentage (log scale) of colonies formed after treatment with indicated doses, corrected according to the plating efficiencies of the corresponding untreated controls, and shown as mean ± S.E.M. (n=3, p-value: EV/2 μM, MOB2= 0.006). **(F)** Survival of control or hMOB2-depleted U2OS cells stably expressing empty vector (EV) or HA- MOB2 following olaparib treatment (AZD-2281, 1 μM, 24h). Results showing the percentage (log scale) of colonies formed after treatment with indicated doses, corrected according to the plating efficiencies of the corresponding untreated controls, and shown as mean ± S.E.M. (n=3, p-value: EV/1 μM, MOB2= 0.009).

**Figure-S5**

Schematic model of hMOB2 involvement in HR.
