# Supplementary material for: hMOB2 deficiency impairs homologous recombination-mediated DNA repair and sensitises cancer cells to PARP inhibitors
Source: Cell Signal. 2021 Nov;87:110106. doi: 10.1016/j.cellsig.2021.110106 (PMC8514680; doi:10.1016/j.cellsig.2021.110106)
Supplement: Supplementary file 2 — Supplementary material 2 [file mmc2.pdf]

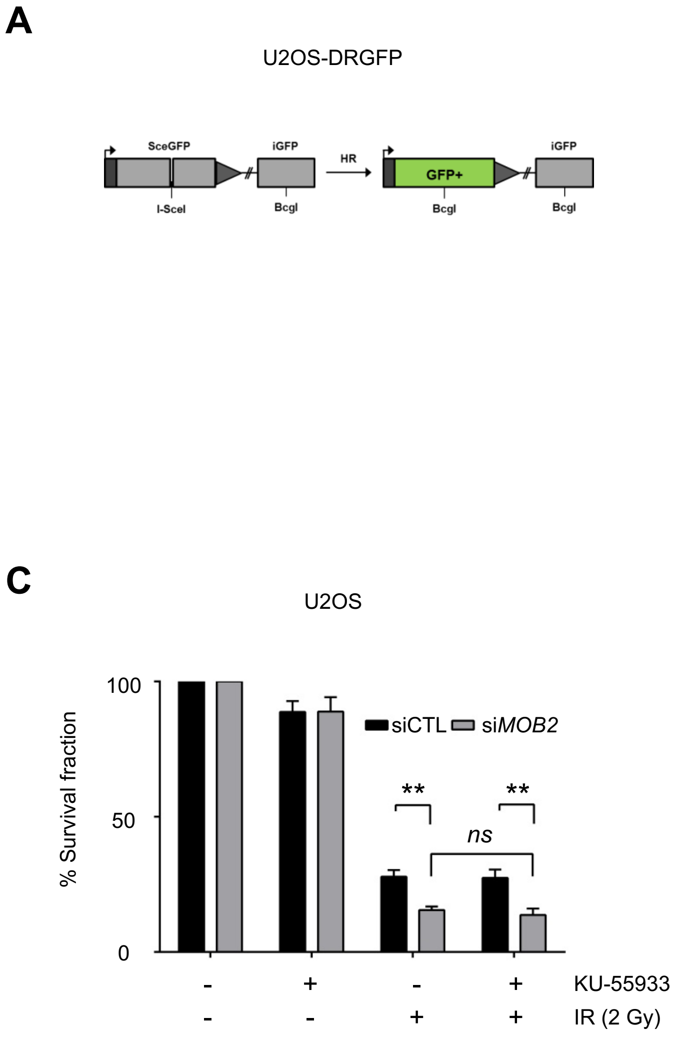

Figure – S1 Gundogdu et al 2021

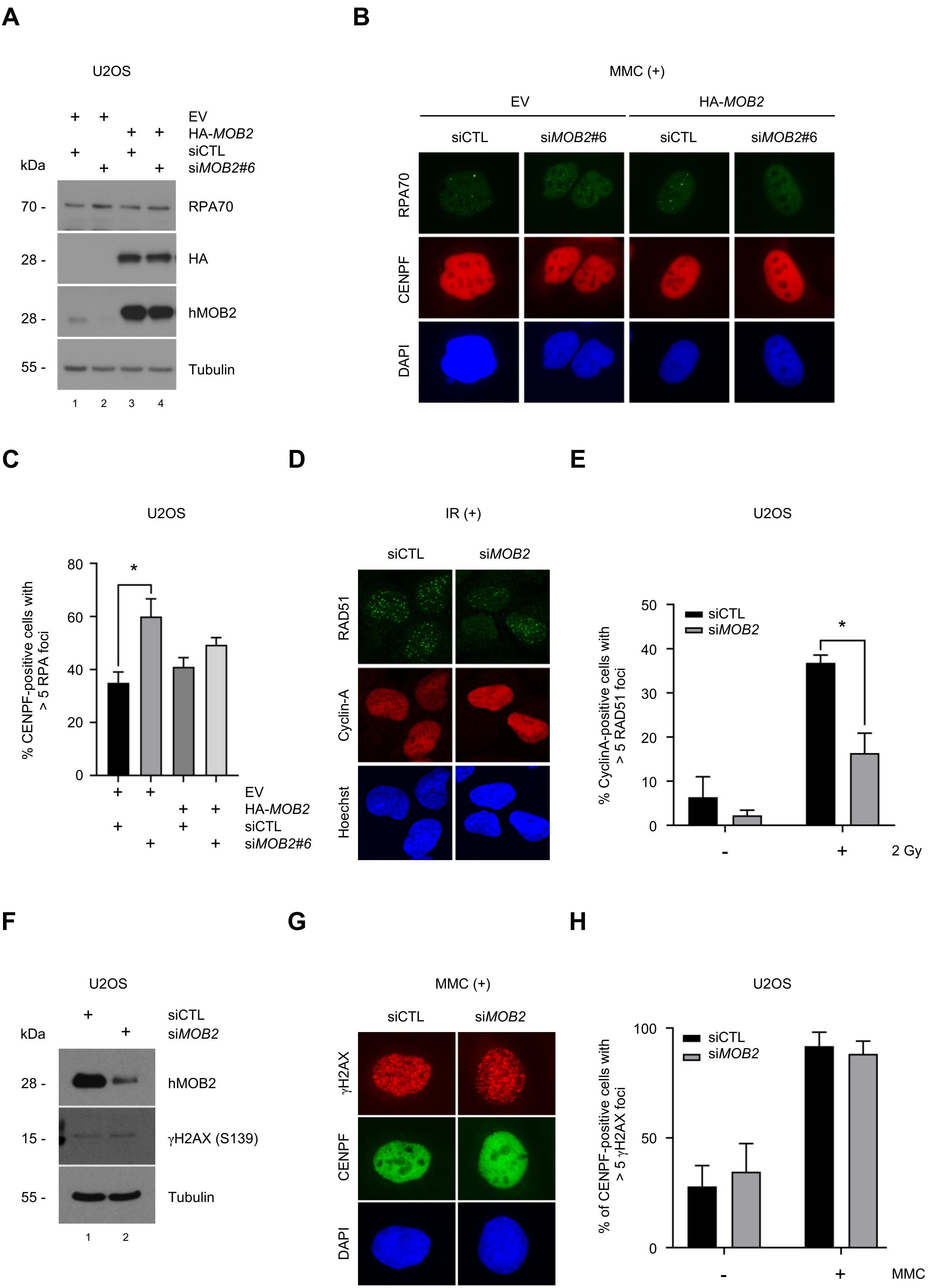

Figure – S2 Gundogdu et al 2021

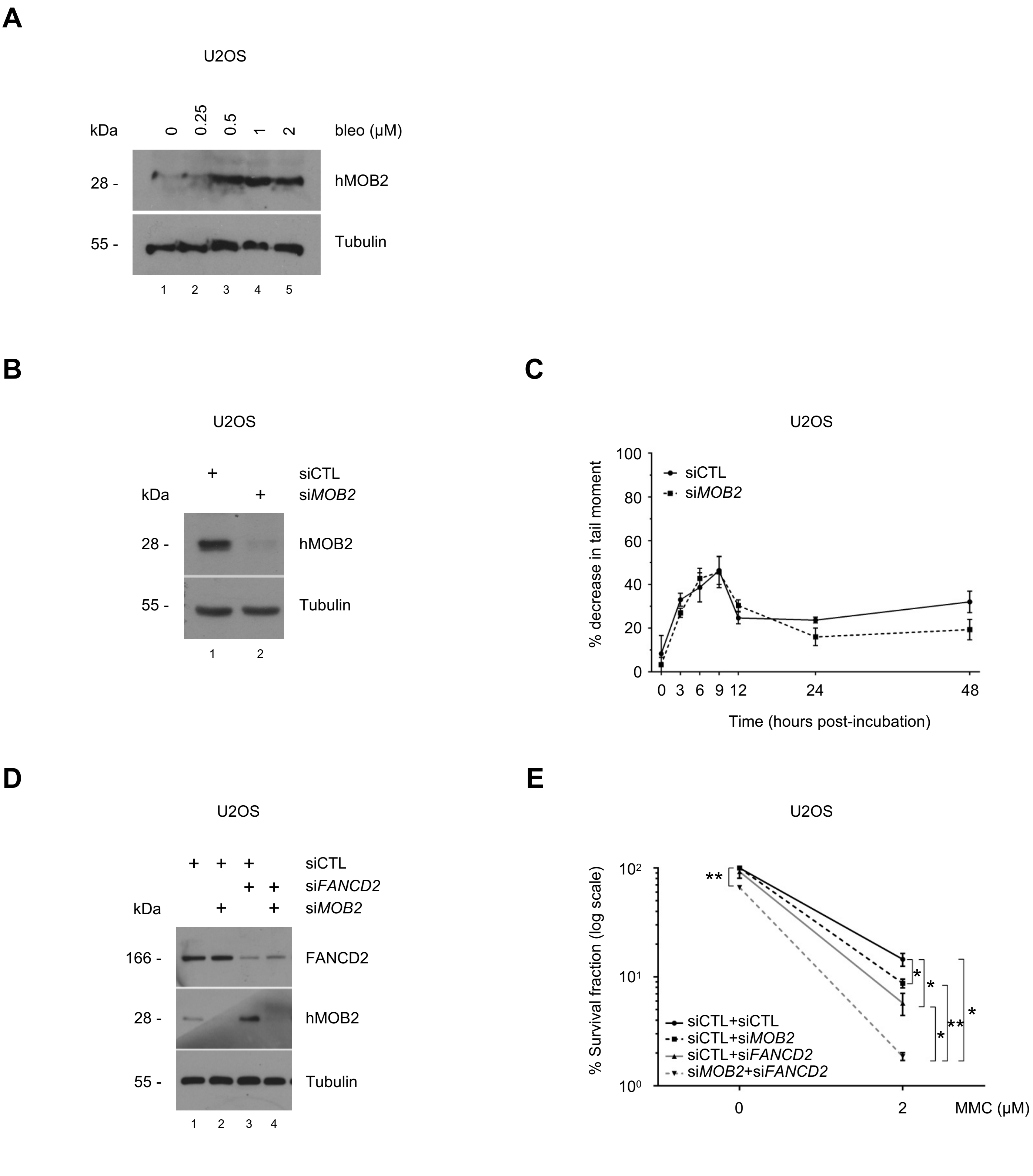

Figure – S3 Gundogdu et al 2021

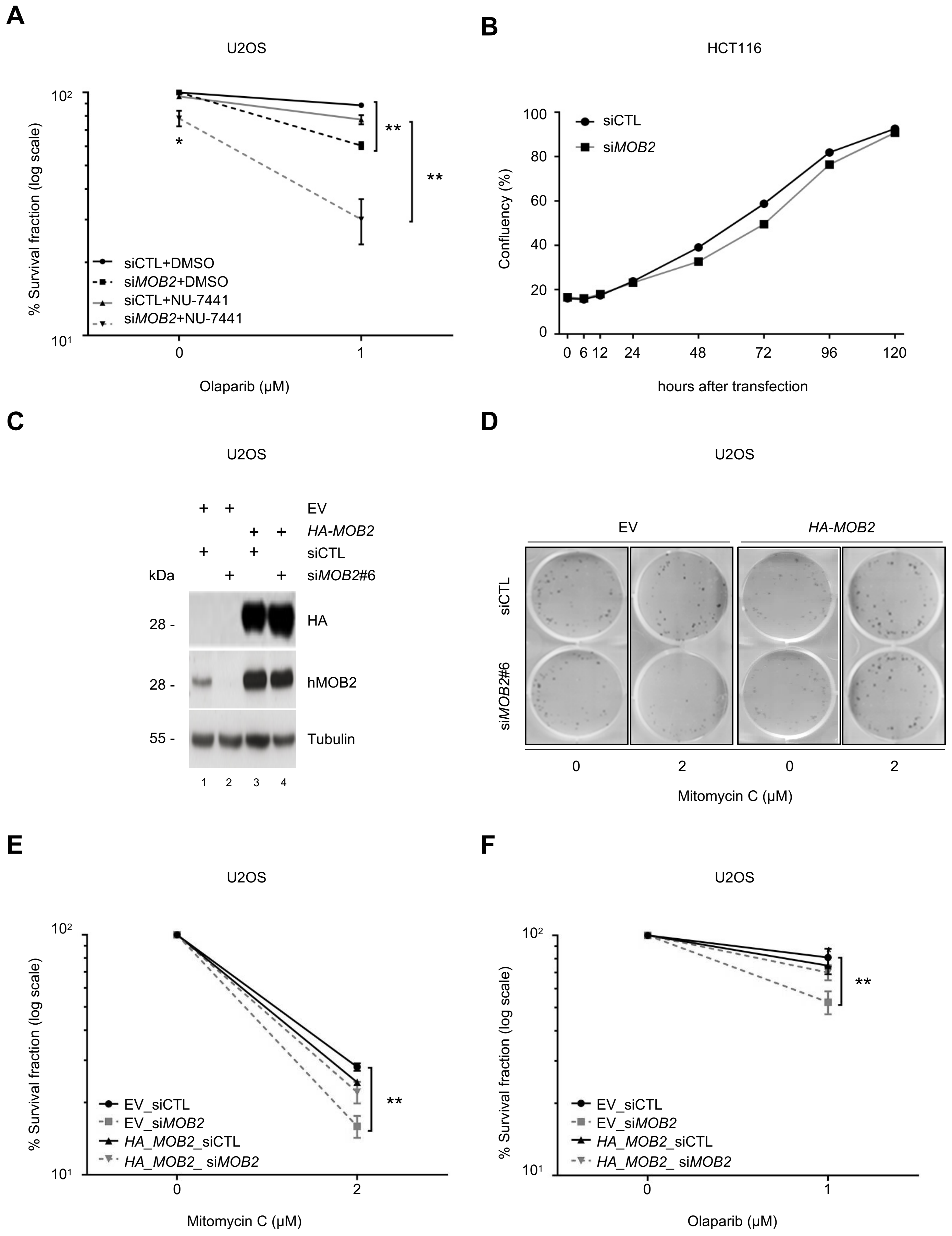

Figure – S4 Gundogdu et al 2021

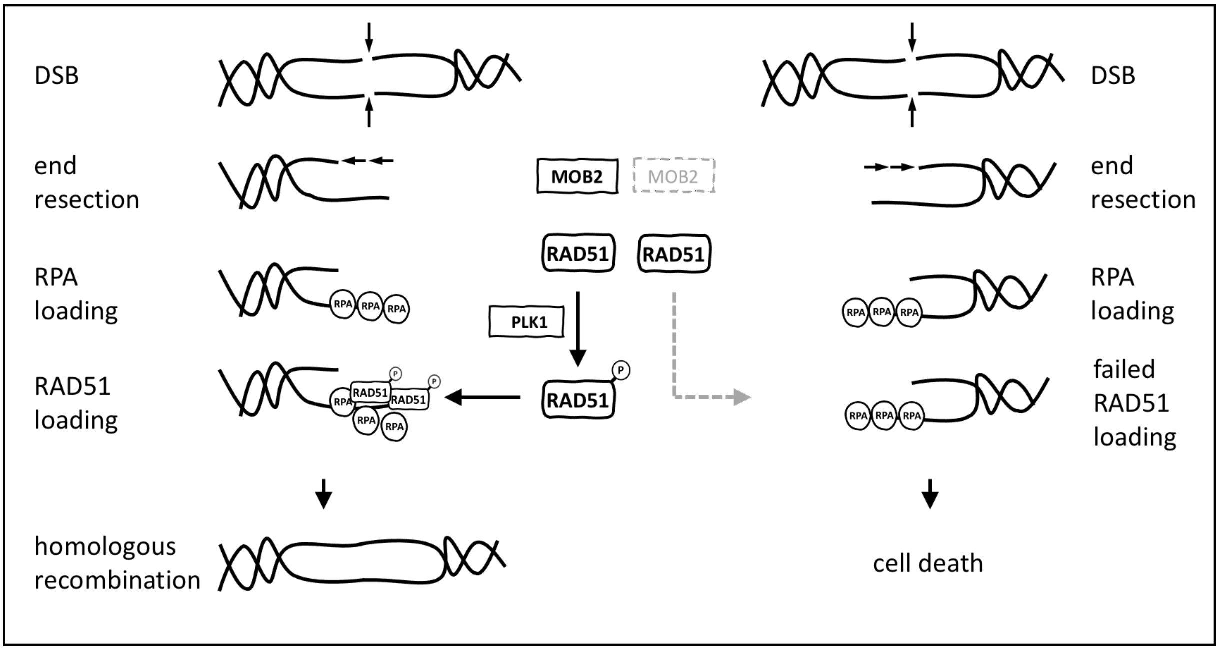

Figure – S5 Gundogdu et al 2021
